# Scanogram leg length measurement after total hip arthroplasty: not all landmarks are created equal

**DOI:** 10.1007/s00256-025-04895-5

**Published:** 2025-02-19

**Authors:** Brian T. N. Le, Yoan Bourgeault-Gagnon, Matthew C. Lyons, Samuel L. McCaffrey, Lucy J. Salmon, Michael D. O’Sullivan

**Affiliations:** 1 North Sydney Orthopaedic and Sports Medicine Centre, Suite 2 The Mater Clinic, 3 Gillies St, Wollstonecraft, Sydney, NSW 2065 Australia; 2 School of Medicine, University of Notre Dame, Sydney, Australia

**Keywords:** Hip arthroplasty, Leg length, Scanogram, Measurement technique, Reliability, Radiology

## Abstract

**Objective:**

To compare the magnitude of post-arthroplasty leg length discrepancy (LLD) and incidence of clinically significant LLD measured on CT scanogram using a commonly used measurement method (from the acetabular apex to tibial plafond) to an alternative technique avoiding the use of the acetabular prosthesis as a landmark and to assess inter-observer and intra-rater reliability of the new technique.

**Materials and methods:**

In this retrospective study, post-arthroplasty LLD measurements were conducted in 100 hips by two interpreters on CT scanogram scout views from the acetabular apex to the tibial plafond (AA-TP) and the inter-teardrop line to the tibial plafond (IT-TP). Aggregate means and proportions of clinically relevant LLD (≥ 10 mm) were compared between methods. Inter-rater reliability was calculated, and both interpreters repeated measurements on ten randomly selected patients to calculate intra-rater reliability.

**Results:**

The commonly used AA-TP technique overestimated LLD by 3.7 mm compared to the IT-TP technique. The odds of LLD measurement exceeding the clinically significant threshold of 10 mm were 3.8 times higher when using the AA-TP technique. Excellent inter-rater (ICC 0.984, 0.958) and intra-rater reliability (ICC > 0.9) were found for both techniques.

**Conclusion:**

CT scanogram measurements from the acetabular apex to the tibial plafond often overestimate operative limb length due to reference landmarks in different axial planes. Measurements from the inter-teardrop line to the tibial plafond yield significantly lower LLD values, possibly reflecting actual limb length better. The authors recommend using the inter-teardrop line and tibial plafond as reference landmarks to improve LLD assessment accuracy post-arthroplasty.

## Introduction

Leg length discrepancy (LLD) after total hip arthroplasty (THA) is a common source of patient dissatisfaction [1–4] and constitutes a very common cause of litigation [5–7], even being reported as the second litigation cause after total hip replacement in the USA [8]. LLD can be divided into ‘true’ LLD (tLLD), where the length difference originates from the intercalated bone segments, and ‘apparent’ LLD (aLLD), which may be caused by hip or knee contractures or altered spinopelvic balance. Around one-third of patients perceive LLD after total hip replacement [1, 9, 10], of which 10–20% are due to a tLLD that can be measured radiologically [9–11]. Clinically, tLLD differences of 10 mm or greater have been associated with worse patient-reported outcomes [9, 12–15].

Numerous radiologic modalities are available to assess LLD, including orthoroentgenogram [9, 16, 17], teleoroentgenogram [17], computed tomography (CT) scanogram [17–20], anteroposterior (AP) pelvis radiographs [17, 21, 22] and EOS scans [23] (EOS Imaging, Paris, France). Full-limb imaging modalities, like CT scanograms, are typically considered the reference standard in the evaluation of suspected LLD. It is essential to recognize the difference between native and post-arthroplasty hip landmarks when evaluating suspected LLD. While CT scanograms assessing primary LLD typically use the superior aspect of the femoral head as the proximal landmark [17, 19, 20], arthroplasty changes the relative position of this landmark in a magnitude that remains unknown. Use of the apex of the acetabular component as the proximal landmark in leg length measurement after hip arthroplasty is common practice in the literature [18], as well as in the authors’ local radiology practices, but is suspected to overestimate the LLD by artificially increasing the measured length of the surgical side. This may have implications both clinically, when counselling patients, and in medicolegal contexts.

Alternative methods for measuring leg length include Woolson’s method [22], which involves measuring the distance between a horizontal line connecting the teardrops (inter-teardrop line) and the lesser trochanters on an AP pelvis radiograph [17, 18, 21, 22]. Interestingly, using the pelvic teardrop as a reference has been shown to be more reliable than other pelvic landmarks [24, 25].

Therefore, this study aimed to evaluate the commonly used leg length discrepancy (LLD) measurement method, which measures from the acetabular apex to the tibial plafond, and to propose an alternative post-arthroplasty technique that avoids using the acetabular prosthesis as a landmark. The primary objective was to compare the magnitude of LLD and the incidence of clinically significant LLD obtained using the standard acetabular apex to tibial plafond (AA-TP) method versus the alternative inter-teardrop line to tibial plafond (IT-TP) technique. The secondary objective was to assess the correlation between LLD measurements from the IT-TP and AA-TP techniques with Woolson’s method. We hypothesized that the AA-TP method would result in a significantly greater LLD than the IT-TP technique, with a higher proportion of patients classified as having clinically significant LLD.

## Materials and methods

This cross-sectional study was approved by an independent ethical board (St. Vincent’s Human Research Ethics Committee, Sydney). Informed consent was obtained from all individual participants included in the study.

### Patients

Consecutive patients who underwent elective total hip arthroplasty at a single private institution by a single surgeon with 20 years of post-fellowship experience (MO) between July 2019 and June 2020 were eligible. All hip arthroplasties were performed using a standard posterolateral approach and a 3D-printed patient-specific instrument to aid in standardizing leg length restoration. The Trinity acetabular prosthesis (Corin Group, Cirencester, UK) was used in all cases. The femoral component used was a mixture of Corail (DePuy Synthes, Warsaw, USA), TaperFit (Corin Group, Cirencester, UK) and MetaFix prostheses (Corin Group, Cirencester, UK).

Patients were excluded if they did not have both a post-operative AP pelvis radiograph and CT scanogram performed at the study institution or if the CT scanogram was of unacceptable quality (apex of acetabular prosthesis or tibial plafond was not captured by the CT scanogram or the lower limb rotation was grossly asymmetrical). Patients undergoing revision surgery, arthroplasty for an oncological lesion and single-stage bilateral arthroplasty were also excluded.

Patients with a contralateral THA performed prior to the study period were included if they were undergoing their second THA during the study period and had an available CT scanogram conducted between their two arthroplasty procedures. In this circumstance, measurements were taken from the CT scanogram performed in between arthroplasty procedures as this was considered equivalent to a post-operative scanogram from the first arthroplasty procedure. This first arthroplasty side was then designated as the “study hip”.

### Leg length discrepancy measurements

Post-operative radiographs and CT scanogram images were taken at the institution’s radiology facility and uploaded onto an Inteleviewer picture archiving and communication system (PACS) (Intelerad Medical Systems, Montreal, Canada) from which digital measurements were taken.

In order to enhance external validity, two interpreters with differing levels of experience in radiograph interpretation (one medical student and one senior resident in orthopaedic surgery) measured tLLD using each of the three techniques (IT-TP, AA-TP and Woolson’s method) on the appropriate imaging modality. Their aggregate mean was utilized as the measurement value in the final dataset for analysis. Standardization meetings were conducted by a fellowship-trained orthopaedic surgeon specializing in hip surgery with reference images illustrating the measurement techniques provided to both interpreters. The first five measurements taken by each interpreter were reviewed by this independent surgeon, and technique modifications were made where required.

**Fig. 1:**
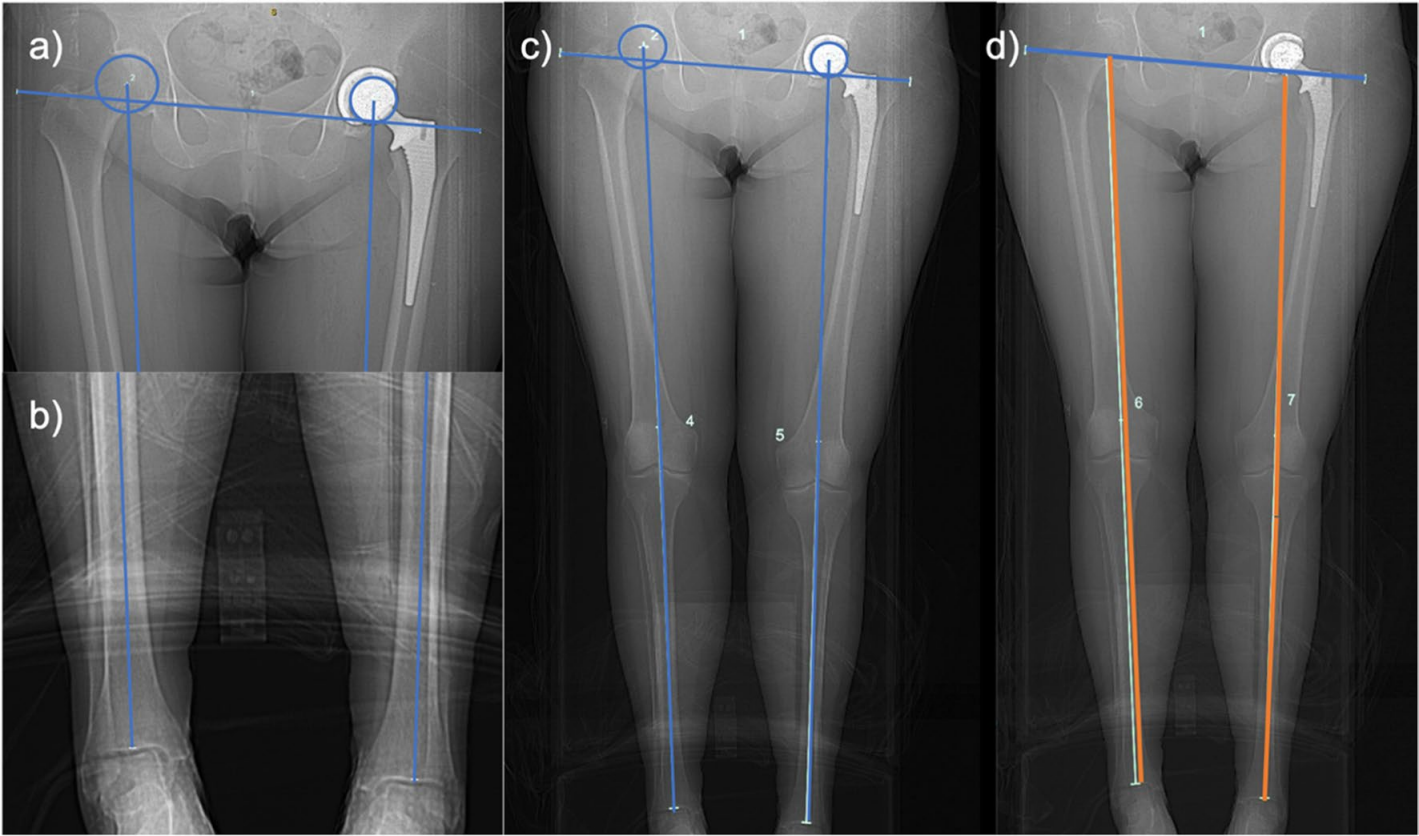
CT scanogram image demonstrating measurement of inter-teardrop to tibial plafond distance (IT-TP). **a** The centre of the native or prosthetic femoral head was defined and a transverse line was drawn passing through the most inferior point of each pelvic teardrop. **b** The centre of the tibial plafond was defined. **c** The mechanical axis of each limb was then drawn between the centres of the femoral head and tibial plafond. **d** The IT-TP (orange line) was defined as the distance between the centre of the tibial plafond and the intersection point of the mechanical axis and inter-teardrop line

The inter-teardrop to tibial plafond distance (IT-TP) was measured on a CT scanogram. The mechanical axis of each limb was defined by a line drawn from the centre of the femoral head (either native or prosthetic) to the centre of the tibial plafond. A separate transverse line was drawn passing through the most inferior point of the pelvic teardrop on each side. The IT-TP was defined as the distance between the centre of the tibial plafond and the intersection point of the mechanical axis and inter-teardrop lines (Fig. 1).

**Fig. 2:**
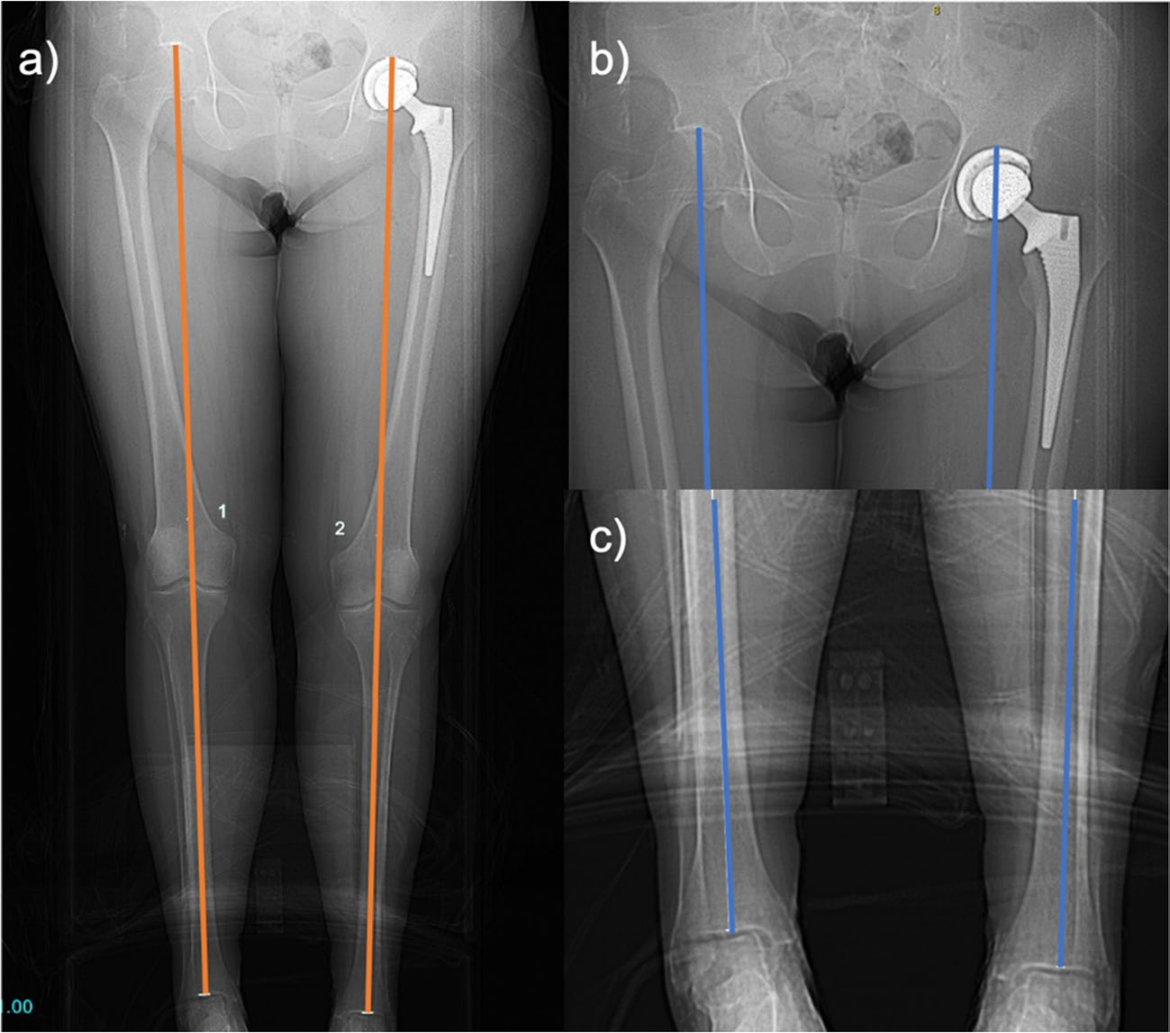
CT scanogram image demonstrating measurement of acetabular apex to tibial plafond distance (AA-TP). **a** The AA-TP (orange line) was defined as the distance between **b** the most proximal aspect of either the acetabular prosthesis or sourcil and** c** the centre of the tibial plafond

The acetabular apex to tibial plafond distance (AA-TP) was measured on CT scanogram as the distance between the centre of the tibial plafond and either the most proximal aspect of either the acetabular prosthesis or the sourcil for native and prosthetic hips, respectively (Fig. 2).

**Fig. 3:**
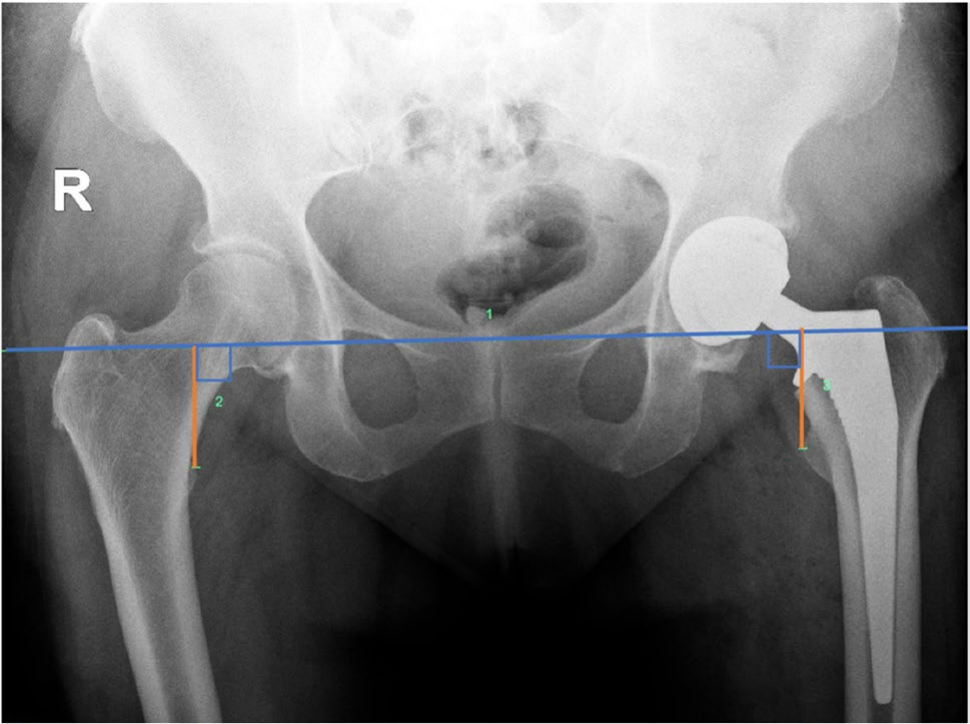
Anteroposterior pelvic radiograph demonstrating measurement of leg length discrepancy according to Woolson’s method which was defined as the perpendicular distance between the inter-teardrop line and the proximo-medial apex of the lesser trochanter (orange line)

Leg length according to Woolson’s method was measured on AP pelvis radiographs as the perpendicular distance between the inter-teardrop line (the above-described transverse line passing through the most inferior point of each pelvic teardrop) and the proximo-medial apex of the lesser trochanter (Fig. 3).

In each case, a positive LLD value was defined as occurring when the study hip limb length was greater than the contralateral limb, whereas a negative value indicated a shorter limb length on the side of the study hip. Measurements were recorded to a 0.1 mm precision.

### Intra-rater and inter-rater reliability measurements

All measurements were performed by two interpreters and were used to calculate inter-rater reliability for each individual measurement method. Both observers repeated LLD measurements using each of the three techniques on ten randomly selected patients 6 months after initial measurements to calculate intra-rater reliability. The observers were blinded to each other’s measurements as well as their own previous measurements during the intra-observer validation stage.

### Statistical analysis

IBM SPSS version 29 was used for the statistical analysis, and all tests were two-tailed with a statistical significance threshold of 0.05.

Continuous outcomes and variables were reported as means (standard deviation [SD]), if normally distributed, or medians (interquartile range [IQR]), if not. Categorial data was reported as proportions.

Since the data was paired and non-parametric, the difference between measurements performed with the IT-TP and AA-TP techniques was evaluated using the Wilcoxon signed-rank test. The proportion of patients in these two groups with a tLLD ≥ 10 mm was compared using McNemar’s test. The absolute risk difference and paired odds ratio were calculated based on the counts of discordant pairs, with 95% confidence intervals computed using standard McNemar’s test methods appropriate for paired categorical data.

The strength of the relationship between the tLLD measured using each of the three previously described techniques was assessed using the Pearson correlation coefficient (*r*) with a 95% confidence interval. The correlation was characterized as poor for *r* < 0.3, fair for *r* 0.3–0.59, moderate for *r* 0.6–0.79 and very strong for *r* > 0.8 [26].

Inter-observer and intra-observer reliability were assessed using the intraclass correlation coefficient (ICC). A two-way mixed effect model with absolute agreement was used for inter- and intra-observer reliability values. The agreement was categorized as slight for ICC < 0.2, fair for 0.21–04, moderate for 0.41–0.6, substantial for 0.61–0.8 and excellent for > 0.8 [27].

## Results

### Included patients

**Fig. 4:**
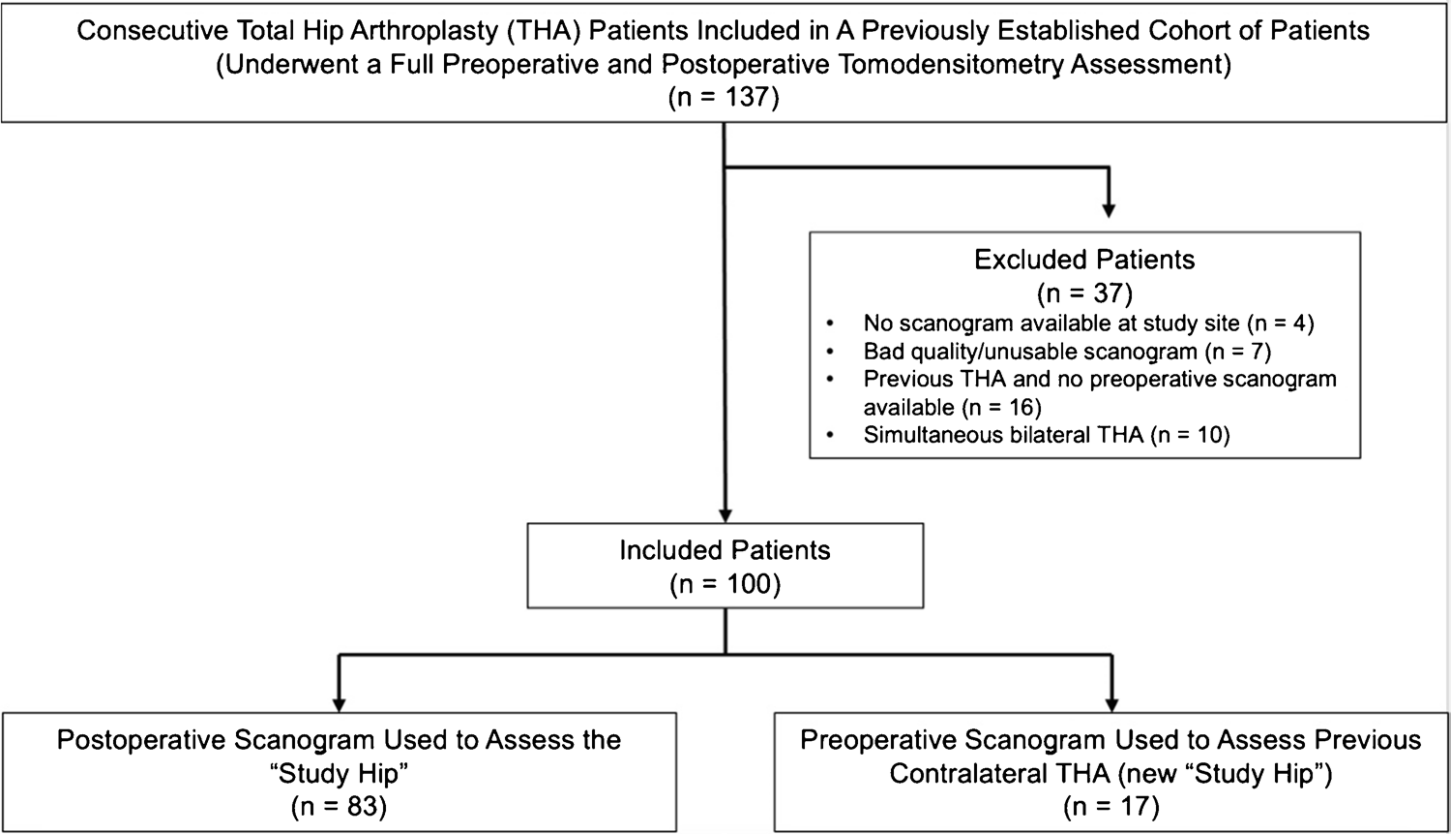
Participant flowchart

One hundred thirty-seven patients underwent elective total hip arthroplasty during the study period, of which 100 patients met inclusion criteria and were included in the final analysis. This included 83 patients whose study hip was replaced during the study period and an additional 17 patients whose study hip arthroplasty occurred prior (Fig. 4). The average age of the participants was 65.4 (range 42–88) with 51 (51%) male and 49 (49%) female patients. There were 73 (73%) right and 27 (27%) left hips assessed. Amongst the 100 study hips, the primary indication for arthroplasty was primary osteoarthritis in 91 hips, developmental hip dysplasia in 6 hips, osteonecrosis in 2 hips and rheumatoid arthritis in 1 hip.

### Leg length discrepancy differences between the inter-teardrop/tibial plafond technique and the acetabular apex/tibial plafond technique

**Fig. 5:**
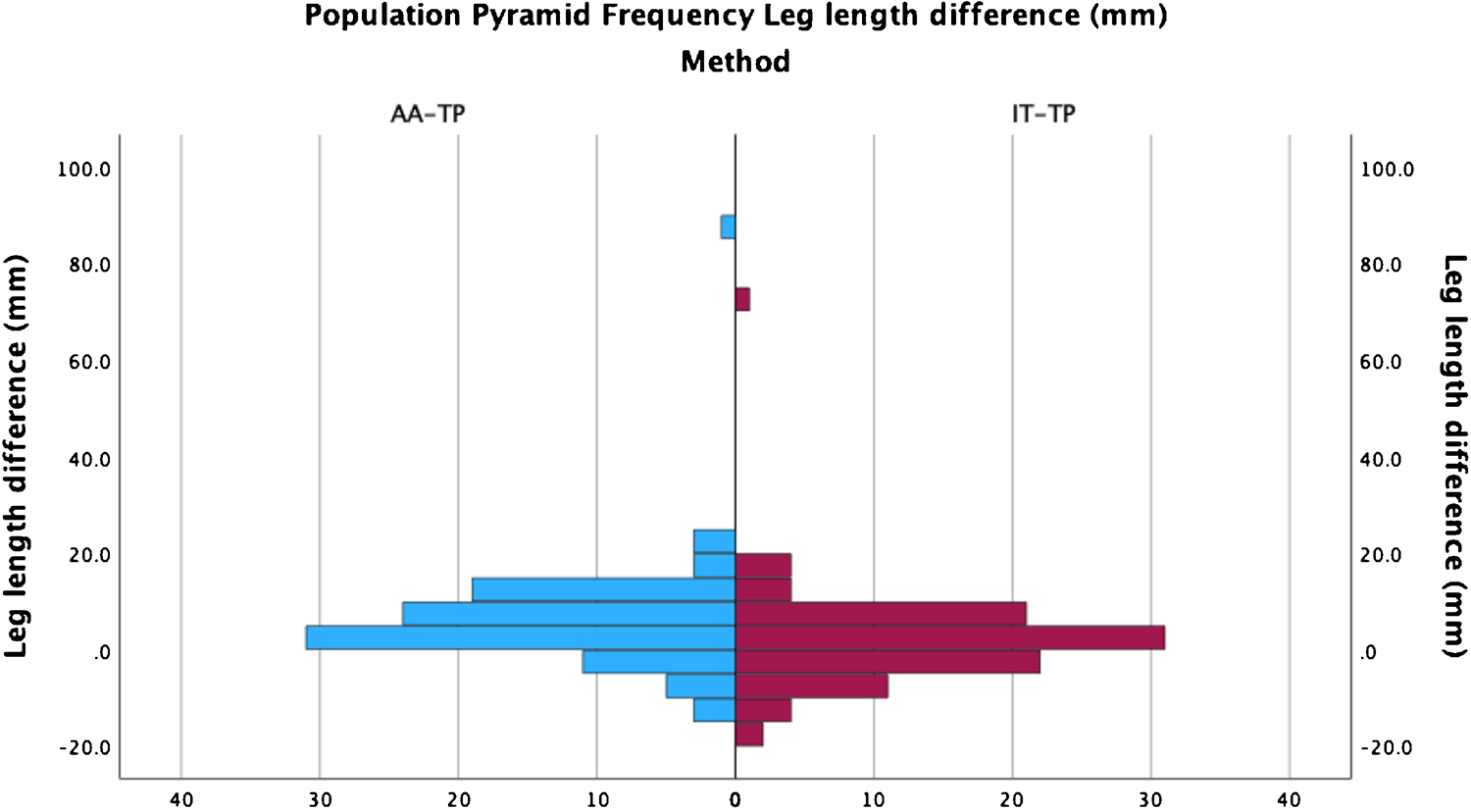
Distribution of leg length discrepancy measurements in the study cohort as measured by the AA-TP and IT-TP techniques

The median leg length discrepancy measured by the IT-TP technique was 1.28 (IQR − 2.99 to 6.83) mm, the AA-TP technique was 4.98 (IQR 1.41 to 10.17) mm and Woolson’s technique was − 0.62 mm (IQR − 3.64 to 3.95) mm. LLD measurements obtained with the AA-TP technique were distributed consistently higher than those obtained with the IT-TP technique (Fig. 5) and significantly greater in magnitude with a median difference of 3.70 mm (*p* < 0.001) (Table 1).

**Table 1:** Comparison of true limb length discrepancy (tLLD) and proportion of patients with clinically significant tLLD

	IT-TP	AA-TP	*p*-value
Median tLLD (mm (IQR))	1.28 (9.8)	4.98 (8.8)	< 0.001
Proportion of patients with tLLD ≥ 10 mm (%)	15	29	0.007

*tLLD* true limb length discrepancy, *IQR* interquartile range, *IT-TP* inter-teardrop to tibial plafond, *AA-TP* acetabular apex to tibial plafond

Fifteen patients (15%) had an LLD of ≥ 10 mm when measurements were made according to the IT-TP technique compared to 29 patients (29%) when measurements were made using the AA-TP technique. A significantly higher percentage of tLLD ≥ 10 mm was found with the AA-TP technique, with an absolute risk difference of 0.14 (95%CI 0.04 to 0.24) and an odds ratio of 3.8 (95%CI 1.42 to 10.18) (*p* = 0.007) (Table 1).

### Measure of association between the three measurement methods

A poor but statistically significant positive correlation was found between LLD as assessed by the AA-TP and Woolson’s methods (*r* = 0.204, 95%CI 0.008 to 0.385, *p* = 0.042). A fair and statistically significant positive correlation was found between LLD as assessed by the IT-TP and Woolson’s methods (*r* = 0.334, 95%CI 0.147 to 0.498, *p* < 0.001). A very strong and statistically significant positive correlation was found between LLD as assessed by the IT-TP and AA-TP methods (*r* = 0.925, 95%CI 0.89 to 0.949, *p* < 0.001).

### Inter-rater and intra-rater reliabilities

Inter-observer correlation was excellent for the IT-TP technique (ICC = 0.958, 95%CI 0.937 to 0.972), AA-TP technique (ICC = 0.984, 95%CI 0.976 to 0.990) and Woolson’s technique (ICC = 0.845, 95%CI 0.763 to 0.897). The intra-rater reliability was excellent for both evaluators across all techniques, with ICC values of 0.985 (95%CI 0.936–0.996) and 0.962 (95%CI 0.853–0.990) for the IT-TP technique, 0.992 (95%CI 0.971–0.998) and 0.982 (95%CI 0.934–0.996) for the AA-TP technique and 0.935 (95%CI 0.773–0.983) and 0.929 (95%CI 0.754–0.982) for Woolson’s technique (Table 2).

**Table 2:** Inter-rater and intra-rater reliability for the three measurement techniques

	Inter-rater reliability ICC (95%CI)	Intra-rater reliability observer 1 ICC (95%CI)	Intra-rater reliability observer 2 ICC (95%CI)
IT-TP	0.958 (0.937–0.972)	0.985 (0.936–0.996)	0.962 (0.853–0.99)
AA-TP	0.984 (0.976–0.99)	0.992 (0.971–0.998)	0.982 (0.934–0.996)
Woolson’s	0.845 (0.763–0.897)	0.935 (0.773–0.983)	0.929 (0.754–0.982)

*IT-TP* inter-teardrop to tibial plafond, *AA-TP* acetabular apex to tibial plafond, *95%CI* 95% confidence interval, *ICC* intraclass correlation coefficient

## Discussion

In the current study, the commonly used AA-TP technique was found to overestimate LLD by 3.70 mm compared to the IT-TP technique. Additionally, there was an absolute increase of 14% in the risk of reporting a clinically significant LLD when the AA-TP technique was used instead of the IT-TP technique. The odds of a result exceeding this clinically significant LLD threshold of 10 mm when using the AA-TP technique were 3.8 times higher than when using the IT-TP technique.

When establishing reference landmarks for leg length, it is essential that they are readily identifiable and exist in the same axial plane to ensure measurement accuracy. This can become challenging in prosthetic joint replacement, where certain anatomical structures may no longer be present bilaterally. For example, using the apex of the acetabular component as the superior landmark on the operative side and either the sourcil or apex of the native femoral head on the contralateral side highlights these issues. Not only do these landmarks exist in different axial planes, but the average distance between them is inconsistent and unpredictable due to factors such as proximalization of the hip joint from acetabular reaming, often resulting in an overestimation of operative limb length.

Routinely assessing LLD after total hip arthroplasty is essential, particularly for patients with dissatisfaction or suboptimal post-operative outcomes [1–3]. Notably, an LLD of 10 mm or more is often associated with clinical issues, including gait asymmetry, back pain and symptomatic or progressive hip and knee osteoarthritis [28–30]. However, overdiagnosis of LLD can also be problematic. The significant increase in reported LLD in this study could theoretically heighten patient anxiety and complicate evaluation in cases of post-operative dissatisfaction. This is especially relevant given the clinical and medicolegal implications of a radiologically measured LLD exceeding published thresholds [5, 6].

The reported rate of clinically significant post-arthroplasty LLD (≥ 10 mm) varies considerably in the literature, with rates between 3 and 23.5% [13, 22]. Other studies have noted mean LLD values of 9–10 mm [1, 9]. Most of these assessments used Woolson’s method exclusively, without a full-limb comparator. There is limited recent data comparing post-operative LLD measured on traditional AP pelvic radiographs to modern full-limb imaging methods. In a study by Hardwick-Morris et al., LLD measurements were conducted using AP pelvic radiographs and EOS scans for 93 patients, revealing a mean LLD of 1.7 mm on AP radiographs (4.2% of the cohort ≥ 10 mm) using Woolson’s method on weight-bearing x-rays, 0.6 mm when measured from the femoral head centre to the ankle centre (17.9% were ≥ 10 mm) and 3.1 mm when measured from the ASIS to the ankle centre (32.6% were ≥ 10 mm) [23]. Although these measurements were pre-operative and cannot be directly compared, the findings, similar to those in this study, underscore the meaningful impact of imaging modality and landmark selection on LLD measurement. To our knowledge, this study is the first to assess the magnitude of LLD differences using various full-limb imaging techniques in the post-operative setting.

Surprisingly, despite the recognized importance of leg length discrepancy after total hip arthroplasty, no widely accepted reference standard exists [31, 32], underscoring the need for studies like this to compare techniques and establish a standard. CT scans are readily accessible in most healthcare settings, and CT scanograms have a high level of accuracy, inter-observer reliability and intra-observer reproducibility when measuring leg length discrepancy [17, 33]. However, other imaging modalities may be preferred in the post-operative follow-up of arthroplasty patients by some practitioners for various reasons, including accessibility, radiation minimization or costs. The authors believe that the findings on measurement techniques from the current study may be applicable to other imaging modalities, particularly other orthogonal modalities like EOS 3D biplanar radiographs (EOS Imaging, Paris, France), which also minimize distortions caused by the parallax effect.

Since many orthopaedic surgeons still use post-operative anteroposterior pelvic radiographs to assess leg length after surgery, the authors wished to compare a commonly utilized technique, Woolson’s technique (distance between inter-teardrop line and lesser trochanter [17, 18, 21, 22]), with both full-limb techniques previously discussed, IT-TP and AA-TP. The degree of correlation between measurements obtained using the IT-TP and Woolson methods, both of which use the inter-teardrop line as their proximal reference line, was only determined to be fair [26]. Woolson’s technique is unable to take into account any factor affecting limb length distal to the lesser trochanter as it is measured on an anteroposterior x-ray of the pelvis only. Additionally, the inter-observer and intra-observer reliability of each technique measured in the current study was categorized as excellent, although Woolson’s method demonstrated the lowest inter-observer agreement of the methods studied. These shortcomings may be also explained by increased variability in lesser trochanter morphology and limb rotation, which can obscure the trochanter behind the femoral shaft, leading to slightly differing interpretations of the lesser trochanter apex location amongst observers. Therefore, in clinical situations where a CT scanogram image is not available, this technique may provide an adequate indication of leg length discrepancy; however, in situations where accuracy is critical, measuring on full-limb imaging, especially using the IT-TP technique, may be preferable.

A strength of the current study is the inclusion of a less experienced interpreter who was a medical student at the time of measurement. The excellent intra-rater and inter-rater reliability results obtained between observers of differing levels of clinical experience enhance the external validity and applicability of our findings.

The current study has some limitations. First, the current project evaluates only the radiological measurements of tLLD, without assessing clinical outcomes or perceived LLD. Therefore, the clinical significance of these findings remains uncertain and requires cautious interpretation. Then, rotation of the lesser trochanter and lower limb was not perfectly controlled for, and seven patients were excluded on the basis of a poor-quality CT scanogram. Whilst this may account for some differences found between measurements taken using the scanogram images and those on AP pelvis x-rays (Woolson’s technique), the IT-TP and AA-TP measurements were made on the same scanogram images.

Current CT scanogram measurements from the acetabular apex to the tibial plafond often overestimate operative limb length due to reference landmarks in different axial planes. Measurements from the inter-teardrop line to the tibial plafond yield significantly lower LLD values, possibly reflecting actual limb length better. The authors recommend using the inter-teardrop line and tibial plafond as reference landmarks to improve LLD assessment accuracy post-arthroplasty.

## Data Availability

The data that support the findings of this study are not openly available due to reasons of sensitivity but are available from the corresponding author upon reasonable request.
